# Higher-level phylogeny of paraneopteran insects inferred from mitochondrial genome sequences

**DOI:** 10.1038/srep08527

**Published:** 2015-02-23

**Authors:** Hu Li, Renfu Shao, Nan Song, Fan Song, Pei Jiang, Zhihong Li, Wanzhi Cai

**Affiliations:** 1 Department of Entomology, China Agricultural University, Beijing. 100193, China; 2 Department of Ornamental Horticulture, China Agricultural University, Beijing. 100193, China; 3 GeneCology Research Centre, Faculty of Science, Health, Education and Engineering, University of the Sunshine Coast, Maroochydore, Queensland, Australia; 4 College of Plant Protection, Henan Agricultural University, Zhengzhou, Henan. 450002, China

## Abstract

Mitochondrial (mt) genome data have been proven to be informative for animal phylogenetic studies but may also suffer from systematic errors, due to the effects of accelerated substitution rate and compositional heterogeneity. We analyzed the mt genomes of 25 insect species from the four paraneopteran orders, aiming to better understand how accelerated substitution rate and compositional heterogeneity affect the inferences of the higher-level phylogeny of this diverse group of hemimetabolous insects. We found substantial heterogeneity in base composition and contrasting rates in nucleotide substitution among these paraneopteran insects, which complicate the inference of higher-level phylogeny. The phylogenies inferred with concatenated sequences of mt genes using maximum likelihood and Bayesian methods and homogeneous models failed to recover Psocodea and Hemiptera as monophyletic groups but grouped, instead, the taxa that had accelerated substitution rates together, including Sternorrhyncha (a suborder of Hemiptera), Thysanoptera, Phthiraptera and Liposcelididae (a family of Psocoptera). Bayesian inference with nucleotide sequences and heterogeneous models (CAT and CAT + GTR), however, recovered Psocodea, Thysanoptera and Hemiptera each as a monophyletic group. Within Psocodea, Liposcelididae is more closely related to Phthiraptera than to other species of Psocoptera. Furthermore, Thysanoptera was recovered as the sister group to Hemiptera.

DNA sequencing and analyses have advanced rapidly in the past decade and the utility of mitochondrial (mt) genomes for phylogenetic inference at various taxonomic levels has been exploited^1^,^2^,^3^,^4^,^5^. Like most other bilateral animals, the mt genomes of insects typically contain 13 protein-coding genes (PCGs), 22 transfer RNA (tRNA) genes, two ribosomal RNA (rRNA) genes, and a large non-coding region (also referred to as the control region, CR)^4^. Analyses of mt genome sequences have improved our understanding of the intraordinal relationships in several insect groups such as Diptera^6^, Orthoptera^7^ and Coleoptera^8^,^9^.

Insect mt genomes tend to have high percentage of A + T content, lineage-specific compositional heterogeneity and accelerated sequence evolution in some groups such as Thysanoptera, Psocodea, Sternorrhyncha (Hemiptera), Strepsiptera, and Hymenoptera^3^,^4^,^10^,^11^,^12^,^13^,^14^. These potential biases limit the applicability of mt genome sequences in the reconstruction of higher-level phylogeny of insects, resulting in incongruence with morphological and nuclear data^3^,^13^,^15^. Among-lineage compositional heterogeneity (e.g., A + T content heterogeneity) and saturation due to accelerated substitution rates are two important processes causing homoplasy in genomic data^16^,^17^. These features, if shared by taxonomically unrelated species, may be responsible for convergent evolution and weaken the true phylogenetic signal^17^. More sophisticated models (e.g., heterogeneous models that allow for heterogeneity across data) that better reflect the evolutionary process and reduce systematic bias are important to phylogenomic study^16^,^18^,^19^,^20^,^21^.

Paraneopteran insects (Acercaria or hemipteroid assemblage) have over 120,400 described species^22^ and are divided into four orders: Hemiptera (aphids, cicadas, planthoppers, true bugs, etc.), Thysanoptera (thrips), Psocoptera (barklice and booklice) and Phthiraptera (parasitic lice)^23^. A large number of paraneopteran insects are agricultural pests, animal parasites and disease vectors^23^. The monophyly of Paraneoptera is widely accepted and supported by morphological, paleontological, molecular, as well as combined morphological and molecular studies^23^,^24^,^25^,^26^,^27^. Although recent phylogenomic studies contradict the widely accepted monophyletic origin of Paraneoptera, but these results are not supported in all statistical tests^28^ or affected by misleading data matrix composition^29^,^30^. At the order level, Hemiptera and Thysanoptera have long been recognized as monophyletic groups^31^,^32^. The monophyly of Psocoptera and Phthiraptera, however, has been challenged in the past several decades. A close relationship between parasitic lice (Phthiraptera) and booklice (Liposcelididae, a family of Psocoptera) was recognized based on morphology^33^. Furthermore, some molecular studies support the close relationship between parasitic lice of the suborder Amblycera and booklice (Liposcelididae)^34^,^35^,^36^. Currently, the superorder Psocodea (= Phthiraptera + Psocoptera) was recognized as being monophyletic whereas its two orders, Phthiraptera and Psocoptera, are mutually paraphyletic^37^.

The higher-level relationships within the Paraneoptera, in particular the position of Thysanoptera, have been controversial for decades. The sister-group relationship between Thysanoptera and Hemiptera, jointly known as Condylognatha, was proposed based on morphological characters^27^,^33^,^38^, ribosomal RNA genes^26^,^35^,^39^ and 1,478 nuclear protein-coding genes^28^. However, studies based on combined molecular and morphological data^24^, and multiple genes^31^, support an alternative sister-group relationship between Psocodea and Thysanoptera. Talavera and Vila (2011) explored the phylogenetic information in mt genomes for inferring interordinal relationships within Paraneoptera, Holometabola and Eumetabola^13^. These authors detected phylogenetic artifacts in all of their datasets; their tree topology was dependent on dataset, phylogenetic algorithm and evolutionary model used. Within the Paraneoptera, a close relationship between Phthiraptera and Thysanoptera was shown in all of their analyses based on limited taxon sampling; these authors suggested that the unexpected grouping of Thysanoptera with Phthiraptera deserved more scrutiny^13^. Here, we included a range of Paraneoptera species much broader than in previous studies and tested: 1) effects of compositional heterogeneity and accelerated substitution rates of mt genome sequences to phylogenetic reconstruction; 2) phylogenetic signals in nucleotide and amino acid datasets; and 3) whether there was a significant improvement in fit with the use of heterogeneous methods over homogeneous models in recovering the higher-level phylogeny of Paraneoptera with mt genome sequences.

## Results

### High degree of compositional heterogeneity

The total A + T content of the PCGs of all included paraneopteran species ranged from 67.71% to 83.27% with a mean of 74.62 (±4.01)%. Within the Psocodea, the A + T content ranged from 67.71% to 78.28% with a mean of 73.29 (±3.75)%. The Thysanoptera had a mean A + T content of 74.97 (±1.02)%, and the Hemiptera had the A + T content from 68.92% to 81.63% with a mean of 75.47 (±4.66)%. Base composition of the PCGs indicated significant heterogeneity in Psocodea and Hemiptera, and between different orders. All nucleotide datasets showed the same pattern and the third codon position of the PCGs had the highest A + T content (Supplementary Fig. S1). Posterior predictive analysis of compositional homogeneity showed that all paraneopteran species in AA dataset, 24 species in PCG dataset and 24 species in PCGRNA dataset were compositionally heterogeneous, further indicating the high degree of compositional heterogeneity in paraneopteran mt genomes (Supplementary Table S1).

### Contrasting substitution rates among paraneopteran mitochondrial genomes

**Figure 1:**
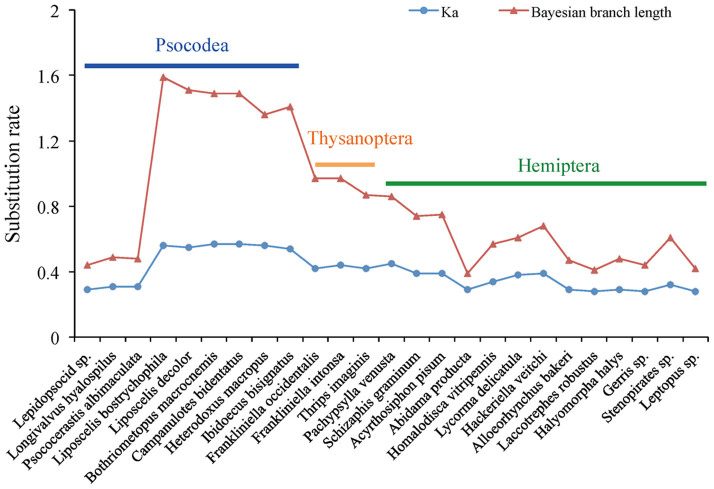
Nucleotide substitution rates among major members of Paraneoptera. *K_a_* was calculated in a pairwise fashion, using *Locusta migratoria* as a reference. Bayesian branch lengths were calculated from the tree of BI-PCGRNA-gene partition, from each taxon to the common ancestor to the Paraneoptera. There is a positive correlation between the result of *K_a_* and Bayesian branch length (R^2^ = 0.97).

We measured *K_a_* (the nonsynonymous substitution rate) for each taxon investigated in this study in comparison with *Locusta migratoria* (Fig. 1, Supplementary Table S2). These comparisons showed that *K_a_* was low for most hemipterans (0.28–0.39) and three barklice (Psocoptera) (0.29–0.31), generally high for Sternorrhyncha (0.35–0.45) and Thysanoptera (0.42–0.44), and extremely high for Liposcelididae (0.55 and 0.56) and Phthiraptera (0.54–0.57). Third codon position of the PCGs had the highest evolutionary rate than the first and second codon positions (Supplementary Fig. S2). Comparison of branch lengths in phylogenetic trees also showed a similar trend (Fig. 1) and a positive correlation was observed between *K_a_* and branch length (R^2^ = 0.97). These results indicate contrasting rates of nucleotide evolution among different paraneopteran lineages, especially a significantly accelerated rate in Phthiraptera, Liposcelididae, Thysanoptera and Sternorrhyncha.

### Heterogeneous sequence divergence within paraneopteran mitochondrial genomes

**Figure 2:**
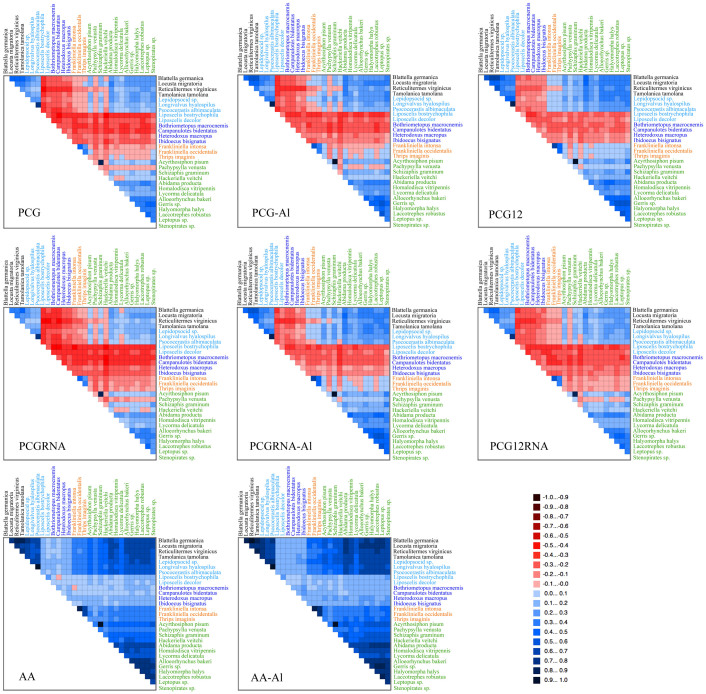
Heterogeneous sequence divergence within paraneopteran mitochondrial genomes. The obtained mean similarity score between sequences was represented by a colored square. The scores were ranging from −1, indicating full random similarity, to +1, non-random similarity. The darker red indicated the higher randomized accordancy between pairwise sequence comparisons. Blue indicated the opposite. All taxa names were listed on top and the right hand side of the matrix with different color, black (outgroup), light blue (Psocoptera), dark blue (Phthiraptera), orange (Thysanoptera) and green (Hemiptera). Dataset name was listed on the bottom left corner and each corresponding abbreviation was clarified in the Methods.

Mt genomes of Paraneoptera showed the high degree of compositional heterogeneity and a significantly accelerated rate in Phthiraptera, Liposcelididae, Thysanoptera and Sternorrhyncha, as indicated in the analyses of base composition and substitution rate. AliGROOVE analyses of various concatenated datasets also found strong heterogeneity of sequence divergence (Fig. 2). For datasets PCG and PCGRNA, pairwise sequence comparisons involving Phthiraptera, Liposcelididae, Thysanoptera and two hemipterans (*Pachypsylla venusta* and *Hackeriella veitchi*) sequences received mainly negative similarity scores while pairwise comparisons between other sequences obtained mainly positive scores. Datasets with data masking (PCG-Al and PCGRNA-Al) and the third codon position excluded (PCG12 and PCG12RNA) decreased the impacts of random sequence similarity and alignment ambiguity for Thysanoptera and two hemipterans whereas Phthiraptera and Liposcelididae still appeared highly divergent with mainly negative similarity scores. Among codon positions of the PCGs, almost pairwise sequence comparisons of the third codon position had negative similarity scores (Supplementary Fig. S3). Amino acid datasets (AA and AA-Al) showed positive similarity scores for nearly all taxon comparisons and the lowest similarity scores for pairwise sequence comparisons involving Phthiraptera and Liposcelididae.

In general, the mt genome sequence datasets of Paraneoptera have strong heterogeneity of sequence divergence and species of Phthiraptera, Liposcelididae and Thysanoptera display mostly random similarity to all other sequences. Cross-validation analyses were performed to test whether there was a significant improvement in fit with the use of heterogeneous models over homogeneous models for datasets. We used the GTR model as a reference to test the fit of CAT and CAT + GTR models for nucleotide dataset, and used MtArt as a reference model for amino acid dataset. As a negative score correspond to a better fit of reference model, results of the cross-validation (all positive scores) indicated that there was strong evidence in favor of heterogeneous models (CAT and CAT + GTR) over the homogeneous models for both nucleotide and amino acid datasets (Table 1). Using the CAT model as a reference, the CAT + GTR model was better fit than the CAT model and thus was the best-fitting model for all datasets (Table 1).

### Phylogeny inferred with maximum likelihood and Bayesian methods using homogeneous models

**Figure 3:**
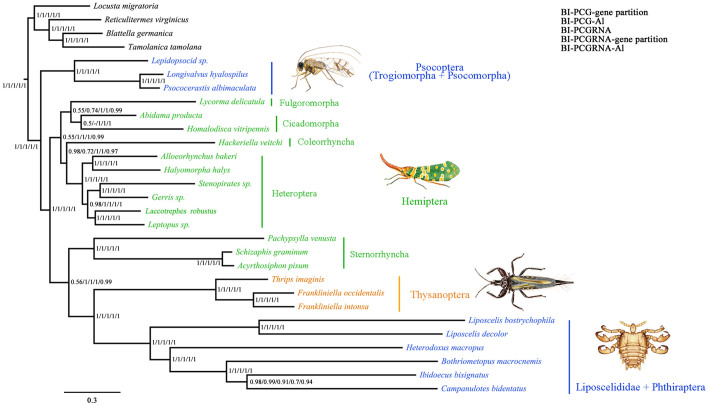
Paraneoptera phylogeny obtained from the Bayesian inferences under homogeneous models. The congruent topology from the analyses of BI-PCG-gene partition, BI-PCG-Al, BI-PCGRNA, BI-PCGRNA-gene partition and BI-PCGRNA-Al. Values at node represented Bayesian posterior probabilities. Results of other methods were shown in Supplementary Fig S4. The illustrations of the four representative paraneopterans were drawn by H. L.

Maximum likelihood (ML) and Bayesian (MrBayes) analyses of the nucleotide and amino acid datasets provided similar topologies for the interordinal relationship of Paraneoptera (Fig. 3, and Supplementary Fig. S4). The monophyly of Psocodea was not recovered, nor the monophyly of Hemiptera. Psocodea was split into two groups: 1) Trogiomorpha and Psocomorpha (suborders of Psocoptera) were together and were sister to the remaining paraneopterans with strong support (bootstrap percentages [BP] > 98 and posterior probabilities [PP] = 1); and 2) Phthiraptera and Liposcelididae were together and were sister to Thysanoptera (BP > 81 and PP = 1). Within Hemiptera, Sternorrhyncha was more closely related to Thysanoptera + (Phthiraptera + Liposcelididae) than to other hemipteran insects (BP > 66 and most PP > 0.93). Bayesian and ML trees from datasets including RNA gene sequences and Bayesian trees from datasets PCG-Al and PCG-gene partition showed better performance and grouped most hemipteran species together, although Hemiptera remained paraphyletic as Sternorrhyncha was sister to Thysanoptera + (Phthiraptera + Liposcelididae) with strong support (BP > 88 and most PP > 0.99) (Fig. 3 and Supplementary Fig. S4a,b). Datasets with the third codon position removed or data masking could not obviously improve the result and produced nearly identical topology of interordinal relationships of Paraneoptera to the corresponding complete dataset.

### Phylogeny inferred with Bayesian method using heterogeneous models

**Figure 4:**
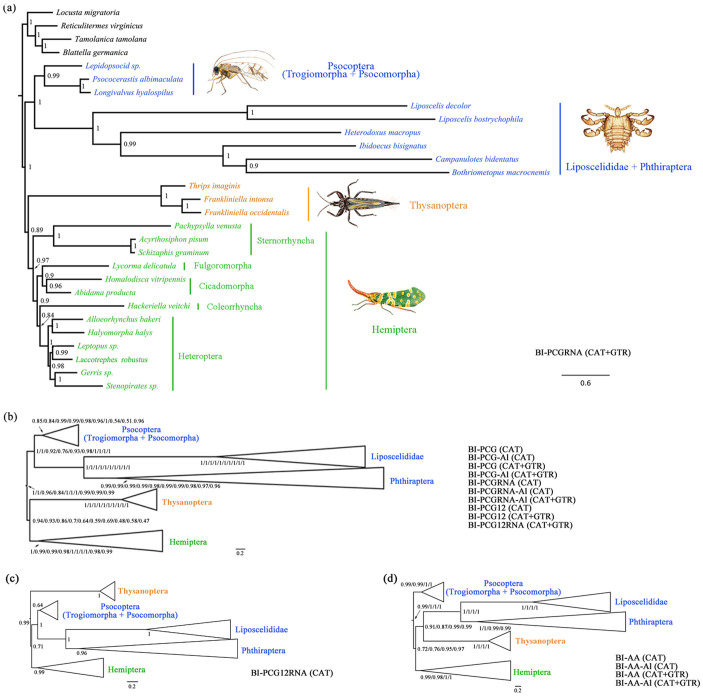
Paraneoptera phylogeny inferred from the PhyloBayes analyses under CAT and CAT + GTR models. (a) The topology from the analysis of BI-PCGRNA-CAT + GTR. (b) The congruent topology from the analyses of BI-PCG-CAT, BI-PCG-Al-CAT, BI-PCG-CAT + GTR, BI-PCG-Al-CAT + GTR, BI-PCGRNA-CAT, BI-PCGRNA-Al-CAT, BI-PCGRNA-Al-CAT + GTR, BI-PCG12-CAT, PCG12-CAT + GTR and PCG12RNA-CAT + GTR. (c) The topology from the analysis of BI-PCG12RNA-CAT. (d) The congruent topology from the analyses of BI-AA-CAT, BI-AA-Al-CAT, BI-AA-CAT + GTR, and BI-AA-Al-CAT + GTR. Values at node represented the Bayesian posterior probabilities. We showed a schematic version of the trees (b–d) with some ingroups collapsed and outgroups removed for clarity. The illustrations of the four representative paraneopterans were drawn by H. L.

Bayesian analyses (PhyloBayes) of all nucleotide datasets using the CAT and CAT + GTR models recovered the monophyly of Psocodea, Thysanoptera, Hemiptera, Phthiraptera and the sister relationship between Phthiraptera and Liposcelididae with high support (most PP > 0.92) (Fig. 4). A sister relationship between Thysanoptera and Hemiptera was also supported by almost nucleotide datasets with Bayesian PP values ranged from 0.47 (BI-PCG12RNA-CAT + GTR) to 0.94 (BI-PCG-CAT) (Fig. 4a,b). Removal of the third codon position of the PCGs seemed to reduce support for the sister relationship between Thysanoptera and Hemiptera, for example, the Bayesian PP values reduced from 0.94 (BI-PCG-CAT) to 0.48 (BI-PCG12-CAT) and from 0.89 (BI-PCGRNA-CAT + GTR) to 0.47 (BI-PCG12RNA-CAT + GTR) (Fig. 4b). Using the dataset PCG12RNA and CAT model, a sister relationship between Hemiptera and Psocodea was supported (PP = 0.71) (Fig. 4c). Results of our slow-fast analyses based on the best fitting CAT + GTR model and nucleotide datasets PCG and PCGRNA showed the similar result that signals supporting Psocodea, Thysanoptera and Hemiptera were stable (Supplementary Fig. S5a,b). However, signal for the sister relationship of Thysanoptera and Hemiptera was concentrated in the fast evolving sites and was lost after removing approximately 50% of the fastest evolving sites. Our results indicated that abundant but competing signals are present in the nucleotide datasets, and the fast evolving sites and the third codon position of the PCGs have useful information for reconstructing interordinal relationship of Paraneoptera based on heterogeneous models, especially for the phylogenetic position of Thysanoptera.

Bayesian analyses of amino acid datasets (AA and AA-Al) using CAT and CAT + GTR models produced four identical topologies (Fig. 4d). The monophyly of Hemiptera was recovered with strong support (PP > 0.98). Psocodea, however, was still paraphyletic. Phthiraptera and Liposcelididae were together, sister to Thysanoptera (PP > 0.87). Trogiomorpha and Psocomorpha were together, sister to all other paraneopteran species (PP > 0.99). Slow-fast analyses showed that signal for the sister relationship of Thysanoptera with Phthiraptera and Liposcelididae was the prevailing one regardless of exclusion of various classes of fast evolving sites in amino acid dataset (Supplementary Fig. S5c). Using CAT-based models and nucleotide datasets, we found that the fast evolving sites and the third codon position of the PCGs have useful information for breaking up the grouping of Thysanoptera with Phthiraptera and Liposcelididae and resolving the phylogenetic position of Thysanoptera. When nucleotide dataset was translated into its corresponding amino acid dataset, these important phylogenetic signals were weakened. Therefore, this explains the poor performance of amino acid data in our phylogenetic analyses based on heterogeneous models.

### Model-based saturation plots and posterior predictive analyses

The phylogenetic effects of homoplasy within individual loci in combined genomic data may strongly bias inferences^5^,^16^,^18^. Compositional heterogeneity and substitutional saturation (multiple substitutions at a single site) are important processes causing homoplasy in genomic data^16^,^17^. To see whether a model is likely to produce artifacts, we can measure how well the model anticipates sequence saturation and homoplasy. If the model does not accommodate them correctly, then it will tend to interpret spurious convergences as true phylogenetic signal and will more likely create artifacts. Considering the different performance of heterogeneous and homogeneous models in tree reconstructions, we used model-based saturation plots and posterior predictive analyses to further test the suitability of these models for resolving the higher-level phylogeny of Paraneoptera.

**Figure 5:**
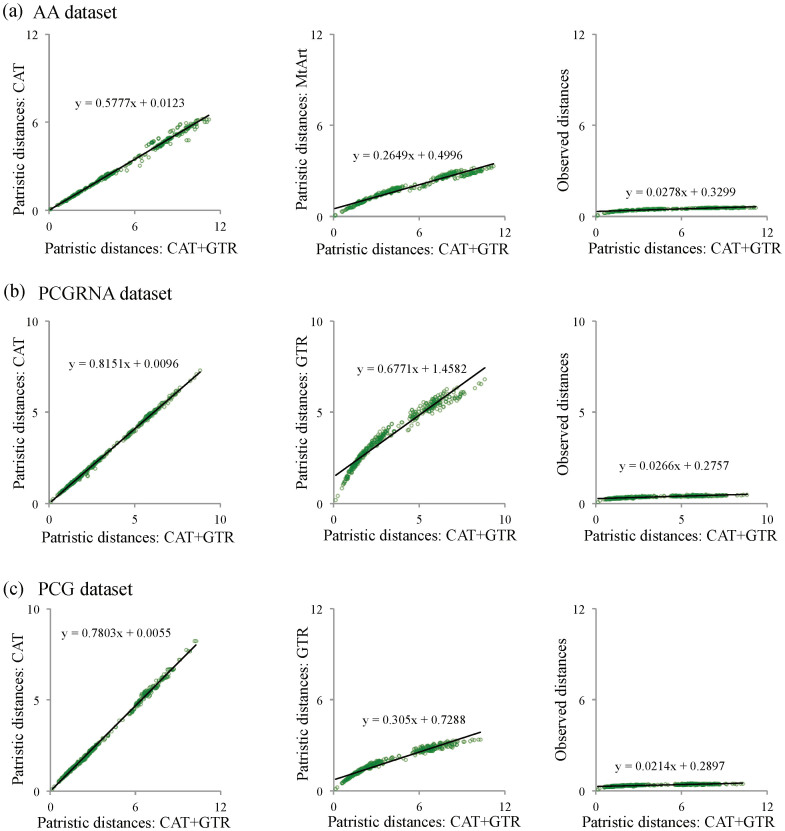
Saturation plots for the amino acid and nucleotide datasets. (a) Plots of the patristic distances of AA dataset estimated from the CAT + GTR tree compared with the distances estimated from the CAT tree and the MtArt tree and the observed distances (uncorrected P-distances). (b) Plots of the patristic distances of PCGRNA dataset estimated from the CAT + GTR tree compared with the distances estimated from the CAT tree and the GTR tree and the observed distances (uncorrected P-distances). (c) Plots of the patristic distances of PCG dataset estimated from the CAT + GTR tree compared with the distances estimated from the CAT tree and the GTR tree and the observed distances (uncorrected P-distances).

Comparisons of saturation plots between CAT + GTR patristic distance and observed distance revealed clear evidence for global substitutional saturation of amino acid (Fig. 5a) and nucleotide (Fig. 5b,c) datasets, with the extremely lower slope (0.0278 for AA, 0.0214 for PCG and 0.0266 for PCGRNA). Saturation plots also showed that the use of heterogeneous and homogeneous models allowed the estimation of trees with comparable patristic distances. When CAT + GTR patristic distances were compared against the corresponding CAT, GTR and MtArt models, it was clear that the GTR and MtArt-based estimations are saturated (Fig. 5). Posterior predictive analyses revealed that MtArt and GTR models inferred a much lower homoplasy than CAT and CAT + GTR models (Supplementary Table S3), and indicated that homogeneous models tend to underestimate homoplasy. On the other hand, heterogeneous models predicted homoplasies in our dataset more efficiently than homogeneous models. These results suggest that trees produced under homogeneous models are likely to display spurious groups. As mentioned above, the grouping of the taxa (Sternorrhyncha, Thysanoptera, Phthiraptera and Liposcelididae) that had accelerated substitution rates and high heterogeneity of sequence divergence was only obtained when the data analyzed under homogeneous models, suggesting that this is a tree reconstruction artifact by using invalid models.

## Discussion

A monophyletic origin of Psocodea is now widely accepted and supported by forewing base structure^38^, attachment structures of the legs^27^ and different types of molecular data (mt *12S* and *16S* rDNA^34^, nuclear *18S* and *28S* rDNA^24^,^26^,^35^,^39^ and 1,478 nuclear protein-coding genes^28^). Phylogenies inferred with concatenated sequences of mt genes, however, failed to recover Psocodea as a monophyletic group but grouped Phthiraptera with Thysanoptera^13^, and Sternorrhyncha with Thysanoptera, Liposcelididae and Phthiraptera in our ML and BI analyses with homogeneous models based on empirical frequencies of amino acid or nucleotide substitutions, like MtArt or GTR-based models. Although the close relationship between Phthiraptera and Liposcelididae has been supported by morphological study^33^ and molecular analyses based on mt *12S* and *16S* rDNA sequences^34^ and a combination of five genes (*18S* rDNA, *H3*, *wingless*, *16S* rDNA and *cox1*)^36^, the grouping of Thysanoptera with Phthiraptera, and Sternorrhyncha with Thysanoptera, Liposcelididae and Phthiraptera has no support from morphological data nor nuclear gene sequences, and therefore is most likely phylogenetic artifacts.

It is clear that heterogeneity in nucleotide composition and substitution rate among the paraneopteran species included in our analysis caused significant substitutional saturation and homoplasy in dataset and complicated the phylogenetic inference. Model-based methods such as likelihood and Bayesian methods suffer from heterogeneity if the assumed model is too simplistic and ignores among-site rate variation^40^,^41^, or gene- and lineage-specific variation in substitution rate and base composition^1^,^21^,^42^,^43^,^44^. Here, the contrasting substitution rates leave Paraneoptera with both short and long branches on phylogenetic trees; this artifact of reconstruction is obvious in phylogenetic analysis under homogeneous models, because the taxa with significantly accelerated substitution rates fall together in one group, e.g., Phthiraptera, Liposcelididae, Thysanoptera and Sternorrhyncha. Most homogeneous models (e.g., GTR and MtArt) assume that: (1) the sequence evolved with the same pattern of nucleotide substitution (homogeneity of the evolutionary process), and (2) all lineages exhibit the same nucleotide composition^42^,^45^,^46^. If these assumptions are not satisfied, as is the case here for paraneopterans with high degree of compositional heterogeneity and substitutional saturation of mt genome sequences, estimation of branch lengths is likely to be biased, which may result in erroneous groupings in the inferred phylogenies.

We applied a variety of strategies in the present study to explore the phylogenetic information in the mt genome sequences of the paraneopteran insects. We found that none of the commonly used methods, e.g., elimination of poorly aligned and divergent positions of genes (e.g., using software trimAl), exclusion of the third codon position of the PCGs, inclusion of rRNA and tRNA genes, data partitioning and using amino acid data, were capable of avoiding erroneous groupings and resolving interordinal relationships of Paraneoptera in our ML and BI analyses with homogeneous models. Indeed, only the Bayesian analysis of nucleotide sequences using heterogeneous CAT and CAT + GTR models was able to separate the long branches and recover the monophyly of Hemiptera and Psocodea, suggesting that the grouping of Sternorrhyncha, Thysanoptera, Liposcelididae and Phthiraptera is a model-dependent tree reconstruction artifact. As the CAT and CAT + GTR models assume the existence of distinct substitution processes and account for compositional heterogeneity in the replacement process^19^,^47^, the use of these models seems to be more effective than homogeneous models and other strategies to predict homoplasies and patristic distances in our dataset, and gives a correct tree.

The monophyly of Condylognatha (Hemiptera and Thysanoptera) was well supported by evidences from morphological characters^27^,^33^,^38^. However, phylogenies based on molecular data have been highly controversial^13^,^24^,^26^,^28^,^31^,^35^,^39^. For example, studies using the *18S* rDNA^35^,^39^, *28S* rDNA^26^ and 1,478 nuclear protein-coding genes^28^ support the monophyly of Condylognatha and the monophyletic Psocodea. Recent phylogenetic analyses (maximum parsimony, ML and BI) based on nucleotide sequences of seven genes (*18S* rDNA, *28S* rDNA, *H3*, *H2A*, *wingless*, *cox1* and *nad4*) and homogeneous models supports the monophyly of Hemiptera but places Thysanoptera as a sister group to Psocodea^35^. In this study, however, Thysanoptera and Psocodea are fast evolving lineages (exhibiting relatively long branch lengths) and their grouping may be due to phylogenetic artifact. Previous phylogeny inferred with CAT model and amino acid sequences of mt 13 PCGs of 17 paraneopteran species including one thrips (Thysanoptera), one barklouse (Psocoptera), two parasitic lice (Phthiraptera), and 13 hemipteran bugs, however, failed to recover Psocodea or Condylognatha as a monophyletic group but grouped Thysanoptera with Phthiraptera^13^. Based on heterogeneous models (CAT and CAT + GTR) and a broader range of taxon sampling, our results found that the third codon position of the PCGs and fast involving sites of mt nucleotide data have useful information for resolving the interordinal relationship of Paraneoptera and the phylogenetic position of Thysanoptera. Bayesian analyses with heterogeneous models and nucleotide datasets including these sites have the high support for the monophyletic Psocodea and the sister relationship of Hemiptera and Thysanoptera. If these sites were excluded or masked, for example using datasets with third codon positions removed or amino acid dataset, support for the sister relationship of Hemiptera and Thysanoptera will be weakened or lost (e.g., Thysanoptera was recovered as the sister to Phthiraptera and Liposcelididae in Bayesian analyses with heterogeneous models and amino acid datasets). Given the variable performance of third codon positions and analysis of the PCGs as amino acids in phylogenetic study of different taxonomic scale^2^,^4^,^5^,^17^,^20^, we suggest that it should be standard practice to assess their effect on topology and nodal support in phylogenetic studies based on mt genome sequences.

Within Psocodea, both morphological and molecular analyses indicate a close relationship between parasitic lice (Phthiraptera) and booklice (family Liposcelididae); the order Psocoptera is therefore paraphyletic^14^,^33^,^34^,^35^,^36^,^49^. Analyses of mt *12S* and *16S* rDNA^34^, nuclear *18S* rDNA^35^ and a combination of mt and nuclear gene sequences (*16S* rDNA, *cox1*, *18S* rDNA, *H3* and *wingless*)^36^ indicate that the parasitic lice are also paraphyletic: the suborder Amblycera is more closely related to the booklouse family Liposcelididae than to the other three suborders of the parasitic lice. Mt genome data, however, support the sister relationship between Phthiraptera and Liposcelididae^14^,^49^. The close relationship between Phthiraptera and Liposcelididae was also strongly supported in all our analyses thus both the monophyly of Phthiraptera and the paraphyly of Psocoptera were supported.

It was worth noting that the contrasting evolutionary rates of mt genomes among psocodean insects resulted in significantly uneven branch length on phylogenetic trees: the extremely long branches in parasitic lice and booklice and the short branches in barklice. To test the possible effect of systematic long-branch attraction (LBA) errors, we used the “long-branch extraction” method^48^ and sequentially removed the rapidly evolving booklice (Liposcelididae) and then the rapidly evolving parasitic lice (Phthiraptera) from our Bayesian analysis of PCGRNA dataset with CAT + GTR model. When the booklice were excluded, the parasitic lice were grouped with the barklice (Trogiomorpha and Psocomorpha) (Supplementary Fig. S6a). When the parasitic lice were excluded, the booklice grouped with the barklice (Supplementary Fig. S6b). The sister relationship of Hemiptera and Thysanoptera was also recovered in both analyses with high supports. Thus, the grouping of the booklice and the parasitic lice cannot be due to LBA because when one of them is removed from the analysis the other remains at the same location on the phylogenetic tree^48^. We should point out that the psocodean taxa included in our analyses are very limited, i.e. three barklice from two families and two booklice from the family Liposcelididae. There are more than 5,700 species in 41 families of the three suborders of Psocoptera (booklice and barklice)^22^. Further analyses with more psocodean taxa in future studies are necessary to validate the conclusions from the present study; the same is true for other paraneopteran lineages.

In summary, it is a challenge to recover the higher-level phylogeny of Paraneoptera with mt genome sequences, due to the significant compositional heterogeneity and the contrasting rates of nucleotide evolution among lineages. We tested a variety of phylogenetic strategies with different datasets of complete mt genome sequences of Paraneoptera species. Our results indicate that: 1) compositional heterogeneity and accelerated substitution rate of mt genome sequences mislead phylogenetic inferences when using invalid models; 2) heterogeneous models (CAT and CAT + GTR) are better than homogeneous models to estimate sequence evolution and reduce phylogenetic errors in mt phylogenomic study; and 3) mt genomes are suitable for resolving higher-level relationships of Paraneoptera but the analysis requires suitable evolutionary model and careful evaluation of which data to include. Our analyses of complete nucleotide sequences (PCG and PCGRNA) using heterogeneous CAT and CAT + GTR models support the following hypotheses: 1) Psocodea is monophyletic; 2) Phthiraptera is monophyletic; 3) Psocoptera is paraphyletic and booklice (Liposcelididae) is more closely related to parasitic lice (Phthiraptera) than to barklice; 4) Thysanoptera is more closely related to Hemiptera than to Psocodea.

## Methods

### Taxon sampling

A total of 29 species of insects were analyzed in this study, including 25 paraneopteran and four outgroup species from the orders Blattodea, Mantodea and Orthoptera. The paraneopteran species are: three barklice (Psocoptera), two booklice (Psocoptera: Liposcelididae), four parasitic lice (Phthiraptera), three thrips (Thysanoptera) and 13 bugs (Hemiptera). Sequences of complete or near complete mt genomes of nine species were generated by us and were published previously^10^,^14^,^49^,^50^,^51^,^52^,^53^,^54^; those of the other species were retrieved from GenBank. Details of the species used in this study were shown in Supplementary Table S4.

### Sequence alignment and dataset concatenation

Sequences of 13 PCGs, two rRNA genes and 17 tRNA genes were used in our analyses. Five tRNA genes that were not available to all sampled paraneopteran species, were excluded: *tRNA^His^*, *tRNA^Asn^*, *tRNA^Met^*, *tRNA^Ala^* and *tRNA^Ser (AGN)^*. PCGs were aligned based on codon-based multiple alignments using the MAFFT algorithm in the TranslatorX online platform^55^ under the L-INS-i strategy and toggled back to the nucleotide sequences. The sequences of tRNA genes were aligned using MXSCARNA^56^ based on the predicted secondary structures. The sequences of two rRNA genes were aligned using Muscle algorithm implemented in MEGA v5.1^57^.

Alignments of individual genes were concatenated as five datasets: 1) PCG: 13 PCGs with 10,749 nucleotides; 2) PCG12: first and second codon positions of 13 PCGs with 7,166 nucleotides; 3) PCGRNA: 13 PCGs, two rRNAs and 17 tRNAs with 14,013 nucleotides; 4) PCG12RNA: first and second codon positions of 13 PCGs, two rRNAs and 17 tRNAs with 10,430 nucleotides; and 5) AA: amino acid sequences of 13 PCGs with 3,583 amino acids. To test the influence of data masking to the phylogenetic analysis, poorly aligned sites were trimmed by using trimAl v1.2^58^ with heuristic automated method. The masked alignments of individual genes were then concatenated as three datasets: 1) PCG-Al: 13 PCGs with 10,269 nucleotides; 2) PCGRNA-Al: 13 PCGs, two rRNAs and 17 tRNAs with 12,106 nucleotides; and 3) AA-Al: amino acid sequences of 13 PCGs with 3,422 amino acids. All eight datasets were used in our phylogenetic analyses.

### Base composition, substitution rate and heterogeneous sequence divergence analyses

Base compositions of different datasets were calculated in MEGA v5.1^57^. Posterior predictive analysis was performed to test whether our dataset included taxa with compositionally heterogeneous sequences by using PhyloBayes v3.3f^59^ under our best fitting CAT + GTR model. We used two measures to compare the degree of substitution rate among paraneopteran species^12^. First, we calculated *K_a_* (the nonsynonymous substitution rate) with DnaSP v5.0^60^. Second, we extracted branch length estimates from the most likely tree after Bayesian analysis of the data. We then manually calculated the branch length for each species, from the tip to the ancestral node for the Paraneoptera. The heterogeneous sequence divergence within dataset was analyzed by using AliGROOVE^61^ with the default sliding window size. Indels in nucleotide dataset were treated as ambiguity and BLOSUM62 matrix was used as default amino acid substitution matrix.

### Testing the fit of homogeneous and heterogeneous models

Cross-validation analyses were performed to test the fit of homogeneous models (GTR and MtArt) and heterogeneous models (CAT and CAT + GTR) to our datasets by using PhyloBayes v3.3f^59^. Cross-validation was calculated by splitting the dataset into two parts, the learning set composed of 90% of the sites in the alignment and a test set composed of the remaining 10% of the sites. A MCMC was run for each learning set, and for each of the compared models, to estimate the parameters of the model. The parameters were then used to calculate the likelihood score of the test set. This was repeated 10 times for each model and the average and standard deviation value of the overall likelihood score was obtained. The scores from each model were then compared and a negative score indicated that the reference model fits the data better than the alternative one.

### Phylogenetic analyses using homogeneous models

We firstly analyzed eight datasets by using both standard Bayesian inference (BI) and maximum likelihood (ML) analysis with homogeneous models. The dataset was not partitioned and the best-fit model was determined using jModelTest 2^62^ for nucleotide and ProtTest 3^63^ for amino acid under AIC, BIC, and AICc criteria. ML analyses were conducted using RAxML- HPC2 v8.1.11^64^ with GTR + I + G model for nucleotide and MtArt + I + G + F model for amino acid, and the reliability of the inferred topology was assessed by performing 500 rapid bootstrap replicates. Bayesian analyses were carried out using MrBayes v3.2.3^65^ with GTR + I + G model for nucleotide dataset and using PhyloBayes MPI v1.4f^47^ with MtArt model for amino acid dataset. For MrBayes, two simultaneous runs of 10 million generations were conducted for the datasets and trees were sampled every 1,000 generations, with the first 25% discarded as burn-in. Stationarity was considered to be reached when the average standard deviation of split frequencies was below 0.01. For PhyloBayes, we run two independent tree searches and stopped them after the likelihood of the sampled trees had stabilized and the two runs had satisfactorily converged (maxdiff less than 0.3).

Three datasets (PCG, PCGRNA and AA) was also used to test the different partitioning schemes for ML and BI methods. The optimal partitioning scheme and substitution model was selected by PartitionFinder v1.1.1^66^. We created input configuration files that contained different predefined partitions for each dataset: 1) 13 gene partitions for PCG (PCG-gene partition); 2) 39 codon partitions for PCG (PCG-codon partition); 3) 32 gene partitions (13 PCGs, 17 tRNAs and two rRNAs) for PCGRNA (PCGRNA-gene partition); 4) 58 partitions (39 codon positions for 13 PCGs, 17 tRNAs and two rRNAs) for PCGRNA (PCGRNA-codon partition); 5) 13 gene partitions for AA (AA-gene partition). We used the ‘‘greedy'' algorithm with branch length estimated as ‘‘unlinked'' and BIC criteria to search for the best-fit partitioning scheme and substitution model. The best selected partitioning schemes and models of three datasets for ML and BI analyses were listed in Supplementary Table S6. Partitioned ML and BI analyses were conducted using RAxML- HPC2 v8.1.11^64^ and MrBayes v3.2.3^65^.

### Phylogenetic analyses using heterogeneous models

Bayesian analyses were also carried out using PhyloBayes MPI v1.4f^47^ with two heterogeneous models, CAT and CAT + GTR, for both amino acid and nucleotide datasets. We run two independent tree searches and stopped them after the likelihood of the sampled trees had stabilized and the two runs had satisfactorily converged (maxdiff less than 0.3). PhyloBayes MPI analyses were conducted in the CIPRES Science Gateway v3.3^67^.

To test the phylogenetic effect of the fast evolving sites in the nucleotide and amino acid datasets, we excluded the fast evolving sites using the SlowFaster^68^. To assign substitution rates to individual positions, three widely recognized groups (Psocodea, Thysanoptera and Hemiptera) were chosen, positions with the highest rates were gradually excluded and new restricted sub-data sets were produced. The nucleotide and amino acid sub-data sets were analyzed with PhyloBayes MPI v1.4f^47^ under CAT + GTR model.

### Model-based saturation plots and posterior predictive analyses

Saturation plots and posterior predictive analyses were used to measure how well the model anticipates sequence saturation and homoplasy. For the saturation plots analyses, the overall best fitting CAT + GTR model was selected as a reference model. Patristic distances derived from trees obtained under other models or using the observed distances (uncorrected P-distances) were plotted against the CAT + GTR distances. The level of saturation was estimated by computing the slope of the regression line in the plot, the lesser the slope, the greater the level of saturation. Patristic distances were generated using PATRISTIC^69^. Posterior predictive analysis implemented in PhyloBayes v3.3f^59^ was used to compare the ability of alternative models to estimate the homoplasy in our datasets.

## Author Contributions

H.L., R.S., Z.L. and W.C. designed and performed the research. H.L., R.S., F.S., N.S. and P.J. analyzed the data. All authors discussed results and implications. H.L., R.S. and W.C. wrote the manuscript. All authors have read and approved the final manuscript.

## Supplementary Material

Supplementary Information Supplementary Figures, Tables and References

## Figures and Tables

**Table 1:** Cross-validation analyses of the homogeneous and heterogeneous models implemented in PhyloBayes based on amino acid and nucleotide datasets

Dataset	Compared models		Cross-validation score	Standard deviation
	Model 1	Model 2		
AA	MtArt	CAT + GTR	213.38	±47.0504
	MtArt	CAT	13.54	±64.6905
	CAT	CAT + GTR	199.84	±20.3261
PCGRNA	GTR	CAT + GTR	639.37	±52.6375
	GTR	CAT	567.69	±53.3731
	CAT	CAT + GTR	71.68	±17.9707
PCG	GTR	CAT + GTR	552.32	±57.6132
	GTR	CAT	489.68	±59.4861
	CAT	CAT + GTR	62.64	±10.8383

Model 1 is the reference model in cross-validation analysis; negative cross-validation score correspond to a better fit of reference model.
