# Supplementary material for: Higher-level phylogeny of paraneopteran insects inferred from mitochondrial genome sequences
Source: Sci Rep. 2015 Feb 23;5:8527. doi: 10.1038/srep08527 (PMC4336943; doi:10.1038/srep08527)
Supplement: Supplementary Information — Supplementary Figures, Tables and References [file srep08527-s1.pdf]

Higher-level phylogeny of paraneopteran insects inferred from mitochondrial genome sequences

Hu Li[1,2]*, Renfu Shao[3]*[§], Nan Song[1,4], Fan Song[1], Pei Jiang[1], Zhihong Li[1], Wanzhi Cai[1§]

[1]Department of Entomology, China Agricultural University, Beijing 100193, China

[2]Department of Ornamental Horticulture, China Agricultural University, Beijing, 100193, China

[3]GeneCology Research Centre, Faculty of Science, Health, Education and Engineering, University of the Sunshine Coast, Maroochydore, Queensland, Australia

[4]College of Plant Protection, Henan Agricultural University, Zhengzhou, Henan 450002, China

* These authors contributed equally to this work.

[§]Correspondence and requests for materials should be addressed to R. S. (rshao@usc.edu.au) and W. C. (caiwz@cau.edu.cn).

Supplementary Information

Supplementary Table S1. Posterior predictive analyses of compositional homogeneity.

	PCG		AA		PCGRNA	
Taxon	p-value	z-score	p-value	z-score	p-value	z-score
Alloeorhynchus bakeri	0	4.515	0	5.783	0	3.65
Frankliniella occidentalis	0	8.312	0	10.677	0	12.146
Heterodoxus macropus	0	13.889	0	4.825	0	17.642
Ibidoecus bisignatus	0	12.24	0	7.866	0	12.176
Liposcelis bostrychophila	0	40.494	0	11.754	0	33.871
Pachypsylla venusta	0.04	2.707	0	5.428	0.1	1.525
Schizaphis graminum	0	166.3	0	63.495	0	180.637
Hackeriella veitchi	0	83.348	0	22.198	0	97.799
Frankliniella intonsa	0	5.011	0	25.447	0	7.171
Liposcelis decolor	0	7.204	0	6.285	0	10.266
Thrips imaginis	0	5.573	0	12.233	0.02	3.353
Abidama producta	0	13.047	0	9.589	0	19.658
Acyrthosiphon pisum	0	170.212	0	64.061	0	189.182

Bothriometopus macrocnemis	0	19.45	0	13.522	0	21.308
Campanulotes bidentatus	0	167.819	0	67.073	0	87.246
Psococerastis albimaculata	0	19.744	0	7.466	0	6.434
Longivalvus hyalospilus	0.7	-0.649	0	9.591	0.11	0.902
Stenopirates sp.	0	158.955	0	48.082	0	109.476
Gerris sp.	0	6.561	0	10.052	0	5.242
Halyomorpha halys	0	4.141	0	4.389	0	5.034
Homalodisca vitripennis	0	22.075	0	7.28	0	15.649
Laccotrephes robustus	0	20.365	0	11.553	0	18.202
Lepidopsocid sp.	0	43.383	0	9.684	0	34.746
Leptopus sp.	0	19.16	0	12.893	0	14.837
Lycorma delicatula	0	10.343	0	25.273	0	35.89
Total number of species with significantly heterogeneous composition		24		25		24

Z-score >2 indicated taxa was significantly compositional heterogeneous.

Supplementary Table S2. Comparison of substitution rates among paraneopteran mitochondrial genomes.

Taxon	K_a	Bayesian branch length	Taxon	K_a	Bayesian branch length
Lepidopsocid sp.	0.29	0.44	*Schizaphis graminum*	0.39	0.74
Longivalvus hyalospilus	0.31	0.49	*Acyrthosiphon pisum*	0.39	0.75
Psococerastis albimaculata	0.31	0.48	*Abidama producta*	0.29	0.39
Liposcelis bostrychophila	0.56	1.59	*Homalodisca vitripennis*	0.34	0.57
Liposcelis decolor	0.55	1.51	*Lycorma delicatula*	0.38	0.61
Bothriometopus macrocnemis	0.57	1.49	*Hackeriella veitchi*	0.39	0.68
Campanulotes bidentatus	0.57	1.49	*Alloeorhynchus bakeri*	0.29	0.47
Heterodoxus macropus	0.56	1.36	*Laccotrephes robustus*	0.28	0.41
Ibidoecus bisignatus	0.54	1.41	*Halyomorpha halys*	0.29	0.48
Frankliniella occidentalis	0.42	0.97	*Gerris* sp.	0.28	0.44
Frankliniella intonsa	0.44	0.97	*Stenopirates* sp.	0.32	0.61
Thrips imaginis	0.42	0.87	*Leptopus* sp.	0.28	0.42
Pachypsylla venusta	0.45	0.86			

Supplementary Table S3. Posterior predictive analyses of sequence homoplasy.

Dataset	Model	Observed Homoplasy	Standard deviation	Predicted Homoplasy	Standard deviation	DH
AA	CAT+GTR	39.5841	3.39281	39.4143	3.40446	0.1698
	CAT	24.8026	0.96474	24.7271	0.965398	0.0755
	MtArt	19.038	0.345349	18.7572	0.477764	0.2808
PCG	CAT+GTR	39.4963	1.48223	39.4715	1.49011	0.0248
	CAT	33.6196	4.46557	33.6346	4.46117	-0.015
	GTR	19.5825	0.414643	19.5531	0.426336	0.0294
PCGRNA	CAT+GTR	34.492	2.22629	34.4639	2.23178	0.0281
	CAT	28.4481	3.23159	28.4706	3.23408	-0.0225
	GTR	17.6091	0.294875	17.5534	0.292151	0.0557

DH is the difference between the observed and the predicted homoplasy, the closer to the zero, the better.

Supplementary Table S4. Species used in this study.

Order/suborder	Family	Species[a]	GenBank	Reference
Isoptera	Rhinotermitidae	*Reticulitermes virginicus*	NC_009500	1
Blattodea	Ectobiidae	*Blattella germanica*	NC_012901	2
Mantodea	Mantidae	*Tamolanica tamolana*	NC_007702	3
Orthoptera	Acrididae	*Locusta migratoria*	JN858206	4
Psocoptera				
Trogiomorpha	Lepidopsocidae	***Lepidopsocid* sp.**	**NC_004816**	**5**
Psocomorpha	Psocidae	***Psococerastis albimaculata***	**NC_021400**	**6**
		Longivalvus hyalospilus	**JQ910986**	**6**
Troctomorpha	Liposcelidae	***Liposcelis bostrychophila***	**JN645275**<br>**JN645276**	**7**
		Liposcelis decolor	NC_023839	8
Phthiraptera				
Ischnocera	Philopteridae	*Bothriometopus macrocnemis*	NC_009983	9
		Campanulotes bidentatus	**NC_007884**	**10**

		Ibidoecus bisignatus	NC_015999	11
Amblycera	Boopidae	***Heterodoxus macropus***	**NC_002651**	**12**
Thysanoptera	Thripidae	***Thrips imaginis***	**NC_004371**	**13**
		Frankliniella occidentalis	NC_018370	14
		Frankliniella intonsa	NC_021378	15
Hemiptera				
Sternorrhyncha	Psyllidae	*Pachypsylla venusta*	NC_006157	16
	Aphididae	*Schizaphis graminum*	NC_006158	16
		Acyrthosiphon pisum	NC_011594	Direct submission
Coleorrhyncha	Peloridiidae	*Hackeriella veitchi*	NC_020309	17
Auchenorrhyncha				
Cicadomorpha	Cicadellidae	*Homalodisca vitripennis*	NC_006899	Direct submission
	Cercopidae	*Abidama producta*	NC_015799	18
Fulgoromorpha	Fulgoridae	*Lycorma delicatula*	NC_012835	19
Heteroptera	Enicocephalidae	***Stenopirates* sp.**	**NC_016017**	**20**
	Gerridae	*Gerris* sp.	NC_012841	19

Nepidae	*Laccotrephes robustus*	NC_012817	19
Leptopodidae	*Leptopus* sp.	FJ456946	19
Nabidae	***Alloeorhynchus bakeri***	**NC_016432**	**21**
Pentatomidae	*Halyomorpha halys*	NC_013272	22

[a] mitochondrial genome sequences of nine species generated from our previous studies were highlighted in bold.

Supplementary Table S5. Best partitioning scheme and model selected by PartitionFinder for phylogenetic analyses.

Dataset and Partition type	Subset Partitions	Model for MrBayes	Model for RAxML
PCG-gene partition	**P1**: (COII, COIII, COI, CYTB, ATP6, ND3)	GTR+I+G	GTR+I+G
	**P2**: (ATP8, ND2, ND6)	GTR+I+G	GTR+I+G
	**P3**: (ND1, ND4, ND4L, ND5)	GTR+I+G	GTR+I+G
PCG-codon partition	**P1**: (COIII_pos1, COII_pos1, COI_pos1, CYTB_pos1)	GTR+I+G	GTR+I+G
	**P2**: (COIII_pos2, COII_pos2, COI_pos2, CYTB_pos2, ATP6_pos2)	GTR+I+G	GTR+I+G
	**P3**: (COIII_pos3, COII_pos3, COI_pos3, CYTB_pos3, ATP6_pos3, ATP8_pos3, ND2_pos3, ND3_pos3, ND6_pos3)	GTR+G	GTR+I+G
	**P4**: (ATP6_pos1, ATP8_pos1, ND1_pos1, ND2_pos1, ND3_pos1, ND4_pos1, ND4L_pos1, ND5_pos1, ND6_pos1)	GTR+I+G	GTR+I+G
	**P5**: (ATP8_pos2, ND1_pos2, ND2_pos2, ND3_pos2, ND4_pos2, ND4L_pos2, ND5_pos2, ND6_pos2)	GTR+I+G	GTR+I+G
	**P6**: (ND1_pos3, ND4_pos3, ND4L_pos3, ND5_pos3)	GTR+G	GTR+I+G
PCGRNA-gene partition	**P1**: (12srRNA, 16srRNA)	GTR+I+G	GTR+I+G

	**P2**: (COII, COIII, COI, CYTB, ATP6, ND3)	GTR+I+G	GTR+I+G
	**P3**: (ATP8, ND2, ND6)	GTR+I+G	GTR+I+G
	**P4**: (ND1, ND4, ND4L, ND5)	GTR+I+G	GTR+I+G
	**P5**: (tRNA-Arg, tRNA-Asp, tRNA-Cys, tRNA-Gln, tRNA-Glu, tRNA-Gly, tRNA-Ile, tRNA-Leu-cun, tRNA-Leu-uur, tRNA-Lys, tRNA-Phe, tRNA-Pro, tRNA-Ser-ucn, tRNA-Thr, tRNA-Trp, tRNA-Tyr, tRNA-Val)	GTR+I+G	GTR+I+G
PCGRNA-codon partition	**P1**: (12srRNA, 16srRNA)	GTR+I+G	GTR+I+G
	**P2**: (COIII_pos1, COII_pos1, COI_pos1, CYTB_pos1)	GTR+I+G	GTR+I+G
	**P3**: (COIII_pos2, COII_pos2, COI_pos2, CYTB_pos2, ATP6_pos2)	GTR+I+G	GTR+I+G
	**P4**: (COIII_pos3, COII_pos3, COI_pos3, CYTB_pos3, ATP6_pos3, ATP8_pos3, ND2_pos3, ND3_pos3, ND6_pos3)	GTR+G	GTR+I+G
	**P5**: (ATP6_pos1, ATP8_pos1, ND1_pos1, ND2_pos1, ND3_pos1, ND4_pos1, ND4L_pos1, ND5_pos1, ND6_pos1, tRNA-Arg, tRNA-Lys, tRNA-Thr, tRNA-Trp)	GTR+I+G	GTR+I+G

	**P6**: (ATP8_pos2, ND1_pos2, ND2_pos2, ND3_pos2, ND4_pos2, ND4L_pos2, ND5_pos2, ND6_pos2)	GTR+I+G	GTR+I+G
	**P7**: (ND1_pos3, ND4_pos3, ND4L_pos3, ND5_pos3)	GTR+G	GTR+I+G
	**P8**: (tRNA-Asp, tRNA-Cys, tRNA-Gln, tRNA-Glu, tRNA-Gly, tRNA-Ile, tRNA-Leu-cun, tRNA-Leu-uur, tRNA-Phe, tRNA-Pro, tRNA-Ser-ucn, tRNA-Tyr, tRNA-Val)	GTR+I+G	GTR+I+G
AA-gene partition	**P1**: (COII, COIII, COI, CYTB, ATP6, ND3)	MtREV+I+G+F	MtArt+I+G+F
	**P2**: (ATP8, ND2, ND6)	MtREV+I+G+F	MtArt+I+G+F
	**P3**: (ND1, ND4, ND4L, ND5)	WAG+I+G+F	MtArt+I+G+F

Figure S1. Base composition as calculated based on nucleotide datasets across paraneopteran insects. PCG, protein-coding genes; PCG1, the first codon position of protein-coding genes; PCG2, the second codon position of protein-coding genes; PCG3, the third codon position of protein-coding genes; PCG12, the combined first and second codon positions of protein-coding genes; PCGRNA, the combined protein-coding genes and RNA genes; PCG12RNA, the combined first and second codon positions of protein-coding genes and RNA genes.

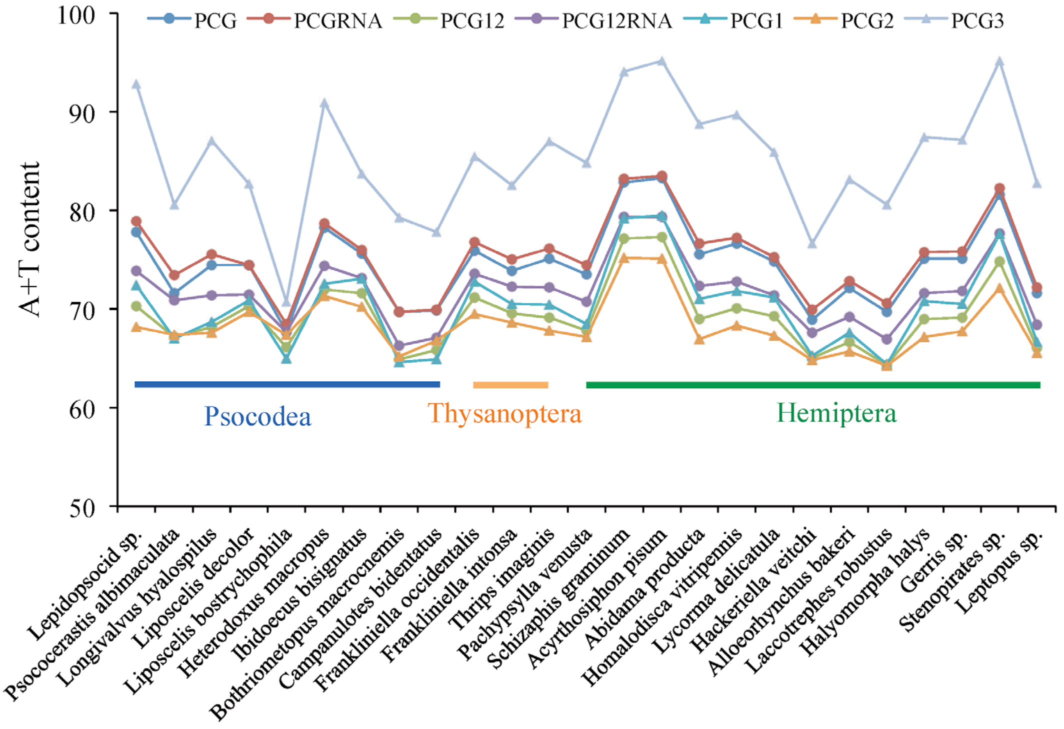

Figure S2. Evolutionary rate of the codon position of protein-coding genes. Genetic distance of each codon position was calculated in a pairwise fashion in MEGA v5.1, using *Locusta migratoria* as a reference.

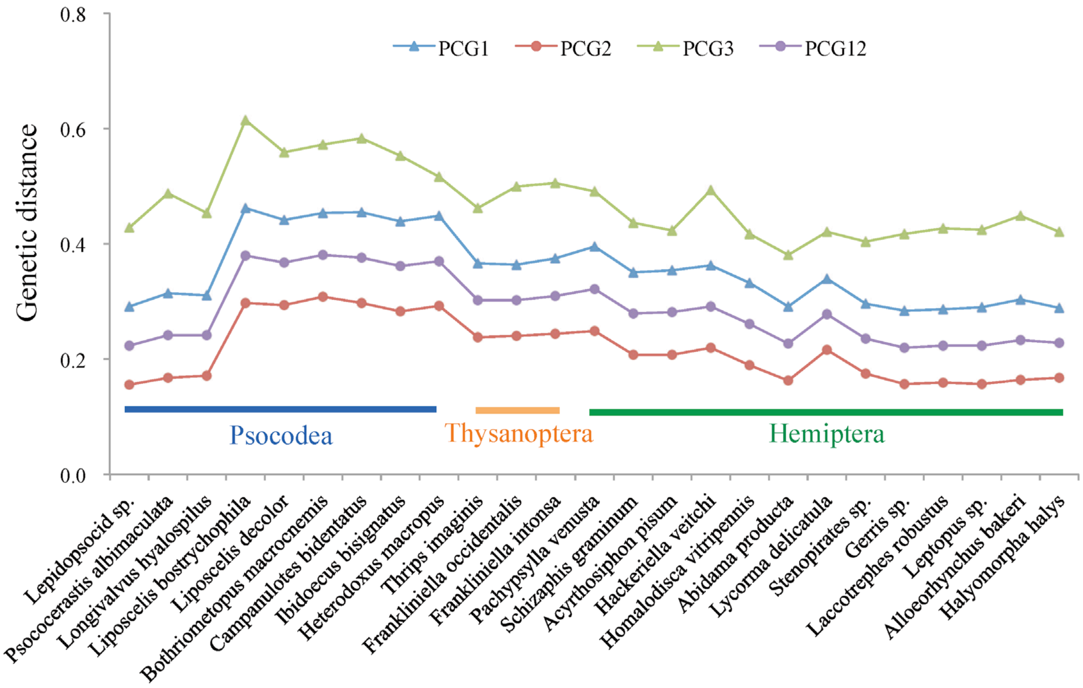

Figure S3. AliGROOVE analyses of the codon position of protein-coding genes. The obtained mean similarity score between sequences was represented by a colored square. The scores were ranging from -1, indicating full random similarity, to +1, non-random similarity. The darker red indicated the higher randomized accordancy between pairwise sequence comparisons. Blue indicated the opposite. All taxa names were listed on top and the right hand side of the matrix with different color, black (outgroup), light blue (Psocoptera), dark blue (Phthiraptera), orange (Thysanoptera) and green (Hemiptera).

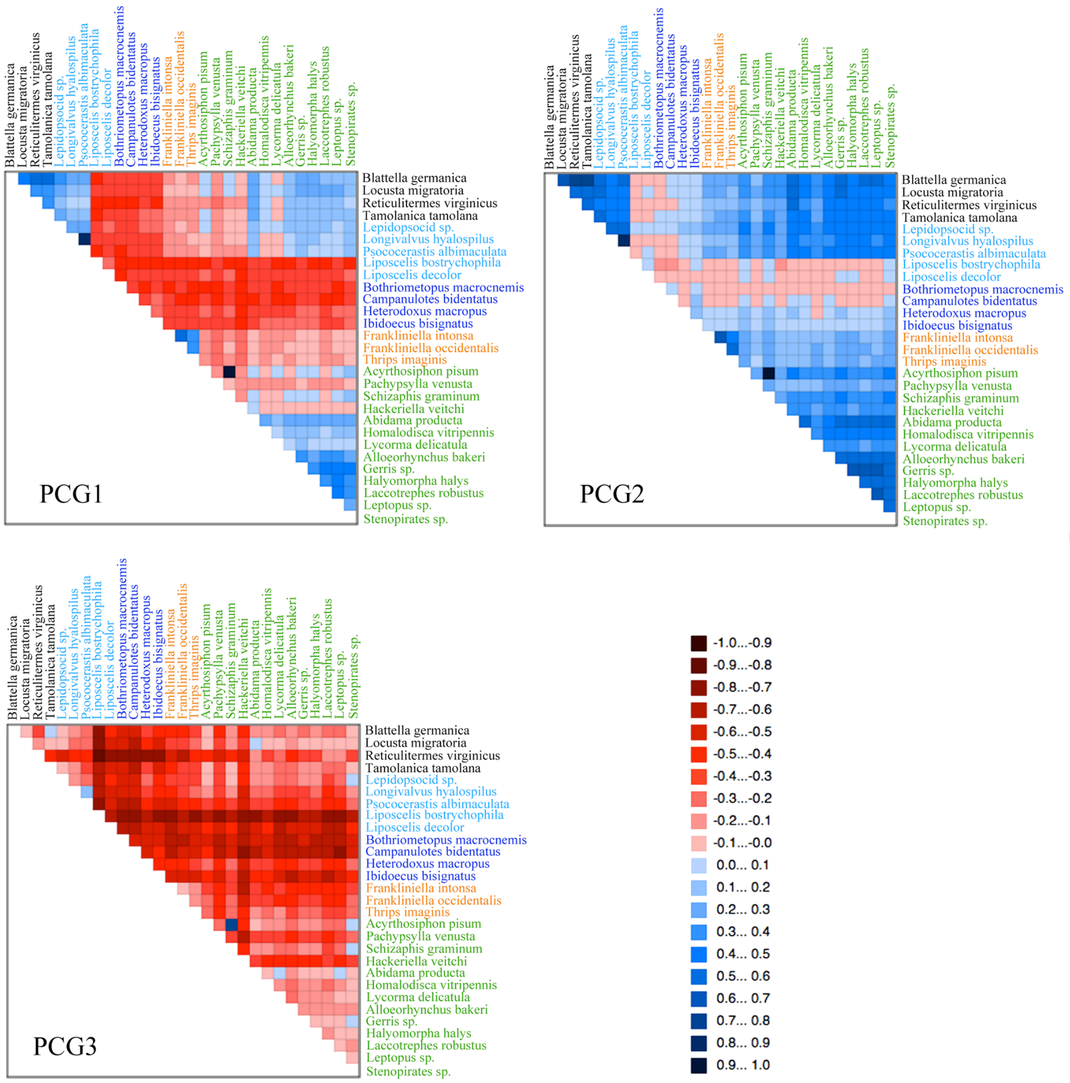

Figure S4. Paraneoptera phylogeny obtained from the Bayesian inference and maximum likelihood analyses under homogeneous models. (a) The congruent topology from the analyses of ML-PCGRNA, ML-PCGRNA-Al and ML-PCGRNA-gene partition. (b) The congruent topology from the analyses of BI-PCGRNA-codon partition, BI-PCG12RNA, ML-PCGRNA-codon partition and ML-PCG12RNA. **(**c) The congruent topology from the analyses of ML-PCG and ML-PCG-Al. (d) The topology from the analysis of BI-PCG. (e) The congruent topology from the analyses of BI-PCG12 and ML-PCG12. (f) The congruent topology from the analyses of BI-PCG-codon partition, ML-PCG-codon partition, BI-AA, BI-AA-Al, BI-AA-gene partition, ML-AA, ML-AA-Al and ML-AA-gene partition. The partitioning schemes and models were listed in Supplementary Table S5. We showed a schematic version of the trees with some ingroups collapsed and outgroups removed for clarity. Values at node represented ML bootstrap percentages and Bayesian posterior probabilities.

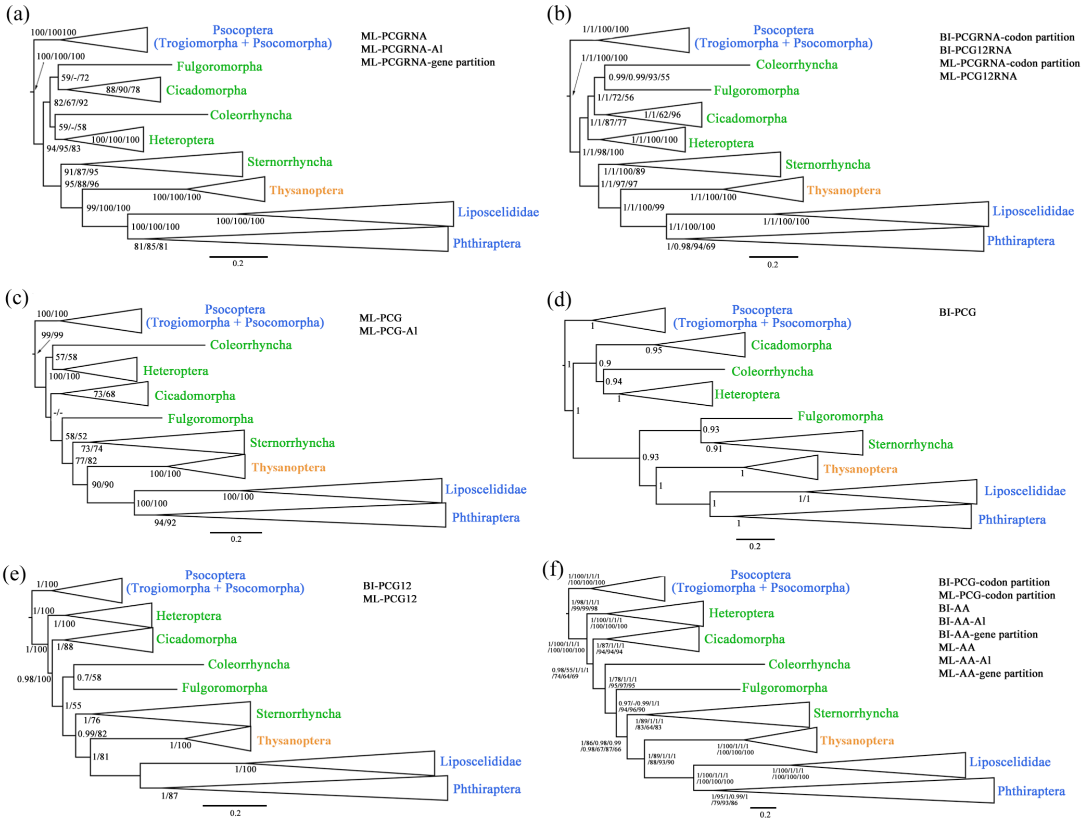

Figure S5. Slow-fast analyses of the nucleotide and amino acid datasets. Posterior probabilities using Bayesian CAT+GTR model for various sub-datasets deprived of classes of fast evolving sites (as indicated by the amount of sites left in the datasets). (a) Slow-fast analyses on PCGRNA dataset. (b) Slow-fast analyses on PCG dataset. (c) Slow-fast analyses on AA dataset. Signals support for Psocodea, Thysanoptera and Hemiptera were high, but support for the sister relationship of Thysanoptera and Hemiptera disappeared after removing about 50% of fastest evolving sites in nucleotide datasets. Signal for the sister relationship of Thysanoptera with Phthiraptera and Liposcelididae was stable in amino acid dataset.

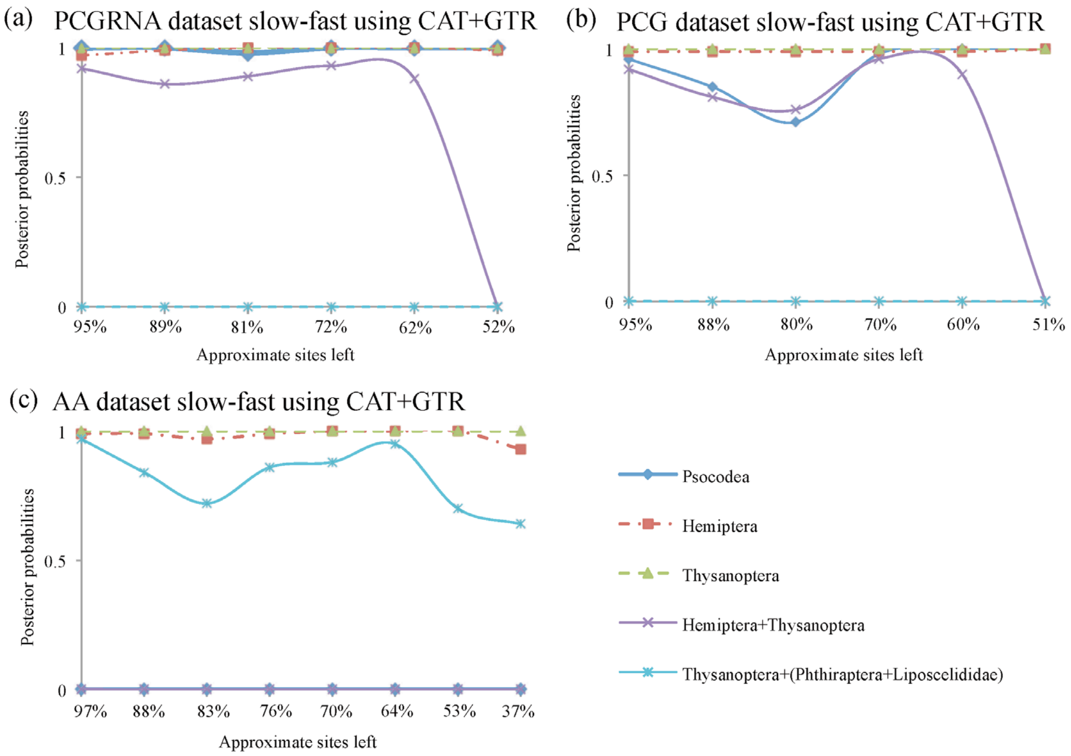

Figure S6. Paraneoptera phylogeny inferred from the PCGRNA dataset with the removal of rapidly evolving lineages using PhyloBayes under CAT+GTR model. (a) The rapidly evolving booklice (Psocoptera: Liposcelididae) were excluded. (b) The rapidly evolving parasitic lice (Phthiraptera) were excluded. Values at node represented Bayesian posterior probabilities. The illustrations of the four representative paraneopterans were drawn by H. L.

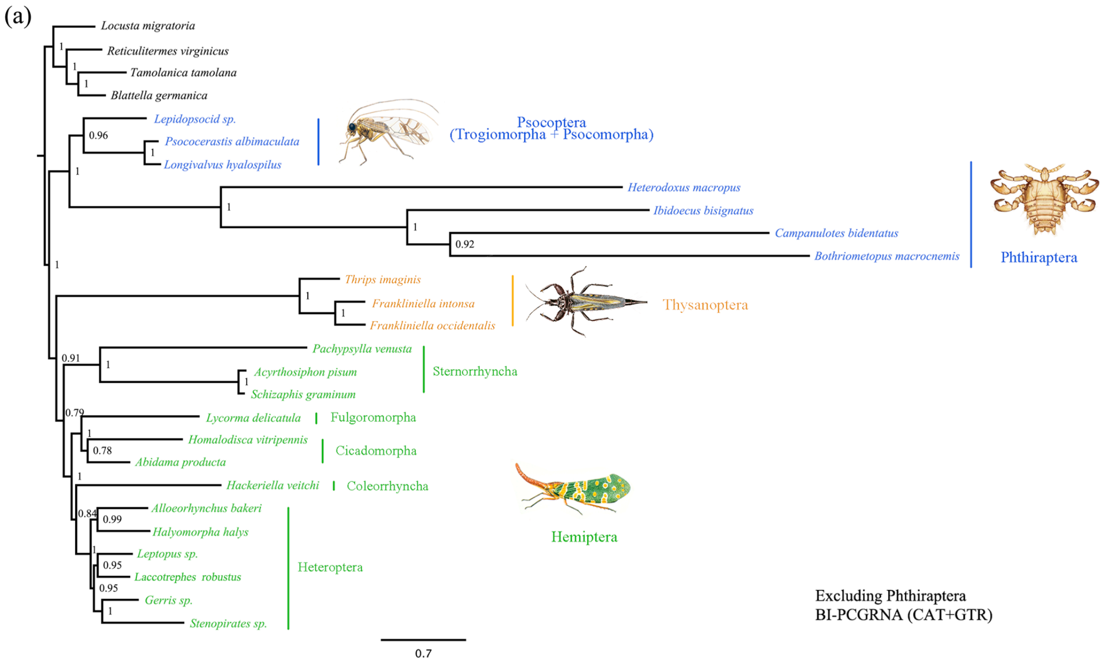

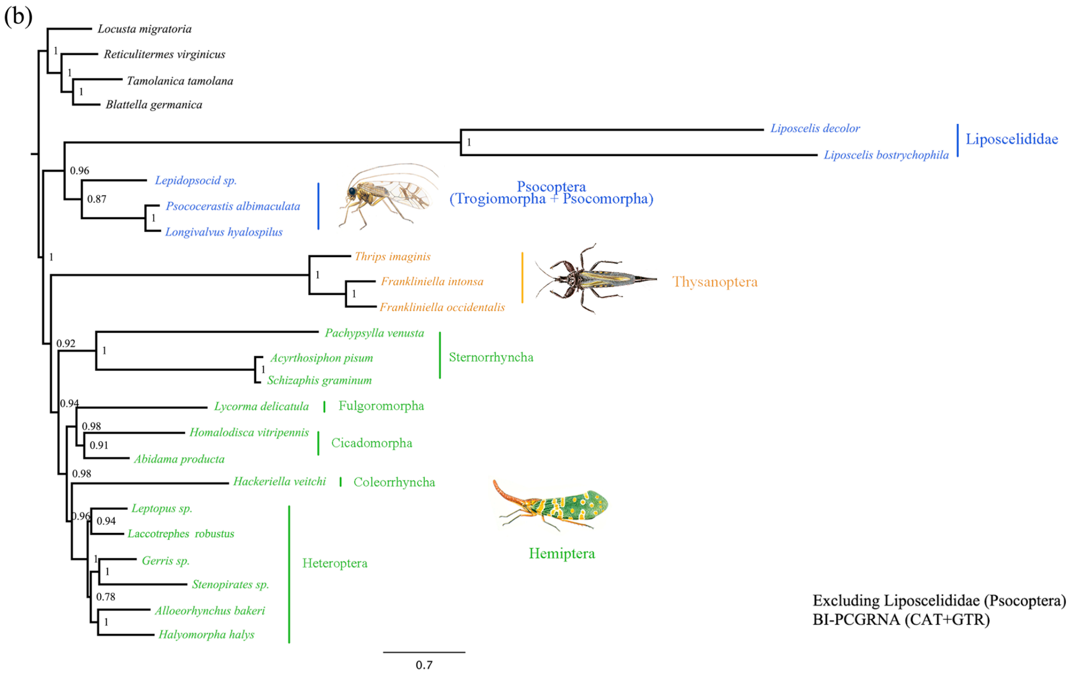

Supplementary References

1. Cameron, S. L. & Whiting, M. F. Mitochondrial genomic comparisons of the subterranean termites from the genus *Reticulitermes* (Insecta: Isoptera: Rhinotermitidae). *Genome* **50**, 188–202 (2007).
2. Xiao, B. *et al*. Complete mitochondrial genomes of two cockroaches, *Blattella germanica* and *Periplaneta americana*, and the phylogenetic position of termites. *Curr. Genet*. **58**, 65–77 (2012).
3. Cameron, S. L., Barker, S. C. & Whiting, M. F. Mitochondrial genomics and the new insect order Mantophasmatodea. *Mol. Phylogenet. Evol*. **38**, 274–279 (2006).
4. Ma, C. *et al*. Mitochondrial genomes reveal the global phylogeography and dispersal routes of the migratory locust. *Mol. Ecol*. **21**, 4344–4358 (2012).
5. Shao, R., Campbell, N. J. H., Schmidt, E. R. & Barker, S. C. Increased rate of gene rearrangement in the mitochondrial genomes of three orders of hemipteroid insects. *Mol. Biol. Evol*. **18**, 1828–1832 (2001).
6. Li, H. *et al.* Mitochondrial genomes of two barklice, *Psococerastis albimaculata* and *Longivalvus hyalospilus* (Psocoptera: Psocomorpha): contrasting rates in mitochondrial gene rearrangement between major lineages of Psocodea. *PLoS ONE* **8**, e61685 (2013).
7. Wei, D. D. *et al*. The multipartite mitochondrial genome of *Liposcelis bostrychophila*: insights into the evolution of mitochondrial genomes in bilateral animals. *PLoS ONE* **7**, e33973 (2012).
8. Chen, S. C., Wei, D. D., Shao, R., Dou, W. & Wang, J. J. The complete mitochondrial genome of the booklouse, *Liposcelis decolor*: insights into gene arrangement and genome organization within the genus *Liposcelis*. *PLoS ONE* **9**, e91902 (2014).
9. Cameron, S. L., Johnson, K. P. & Whiting, M. F. The mitochondrial genome of the screamer louse *Bothriometopus* (Phthiraptera: Ischnocera): effects of extensive gene rearrangements on the evolution of the genome. *J. Mol. Evol*. **65**, 589–604.

10. Covacin, C., Shao, R., Cameron, S. & Barker, S. C. Extraordinary number of gene rearrangements in the mitochondrial genomes of lice (Phthiraptera: Insecta). *Insect Mol. Biol.* **15**, 63–68 (2006).

11. Cameron, S. L., Yoshizawa, K., Mizukoshi, A., Whiting, M. F. & Johnson, K. P. Mitochondrial genome deletions and minicircles are common in lice (Insecta: Phthiraptera). *BMC Genomics* **12**, 394 (2011).

12. Shao, R., Campbell, N. J. H. & Barker, S. C. Numerous gene rearrangements in the mitochondrial genome of the wallaby louse, *Heterodoxus macropus* (Phthiraptera). *Mol. Biol. Evol.* **18**, 858–865 (2001).

13. Shao, R. & Barker, S.C. The highly rearranged mitochondrial genome of the plague thrips, *Thrips imagines* (Insecta: Thysanoptera): convergence of two novel gene boundaries and an extraordinary arrangement of rRNA genes. *Mol. Biol. Evol.* **20**, 362–370 (2003).

14. Yan, D. K. *et al.* The complete mitochondrial genome sequence of the western flower thrips *Frankliniella occidentalis* (Thysanoptera: Thripidae) contains triplicate putative control regions. *Gene* **506**, 117–124 (2012).

15. Yan, D. K. *et al.* The mitochondrial genome of *Frankliniella intonsa*: insights into the evolution of mitochondrial genomes at lower taxonomic levels in Thysanoptera. *Genomics* **104**, 306–312 (2014).

16. Thao, M. L., Baumann, L. & Baumann, P. Organization of the mitochondrial genomes of whiteflies, aphids, and psyllids (Hemiptera: Sternorrhyncha). *BMC Evol Biol.* **4**, 25 (2004).

17. Cui, Y. *et al.* Phylogenomics of Hemiptera (Insecta: Paraneoptera) based on mitochondrial genomes. *Syst. Entomol.* **38**, 233–245 (2013).

18. Liu, J., Bu, C., Wipfler, B. & Liang, A. Comparative analysis of the mitochondrial genomes of Callitettixini Spittlebugs (Hemiptera: Cercopidae) confirms the overall high evolutionary speed of the AT-rich region but reveals the presence of short conservative elements at the tribal level. *PLoS ONE* **9**, e109140 (2014).

19. Hua, J. M. *et al*. Phylogenetic analysis of the true water bugs (Insecta: Hemiptera: Heteroptera: Nepomorpha): evidence from mitochondrial genomes. *BMC Evol. Biol.* **9**, 134 (2009).
20. Li, H. *et al*. The complete mitochondrial genome and novel gene arrangement of the unique-headed bug *Stenopirates* sp. (Hemiptera: Enicocephalidae). *PLoS ONE* **7**, e29419 (2012).
21. Li, H. *et al*. Comparative mitogenomic analysis of damsel bugs representing three tribes in the family Nabidae (Insecta: Hemiptera). *PLoS ONE* **7**, e45925 (2012).
22. Lee, W. *et al*. Complete mitochondrial genome of brown marmorated stink bug *Halyomorpha halys* (Hemiptera: Pentatomidae), and phylogenetic relationships of hemipteran suborders. *Mol. Cells* **28**, 155–165 (2009).
